# Acute Chest Pain Following Bravo Device Placement

**DOI:** 10.7759/cureus.43995

**Published:** 2023-08-23

**Authors:** Ruha Reddy, Brian A Holliday, Marna Greenberg, Gavin C Barr

**Affiliations:** 1 Department of Emergency and Hospital Medicine, Lehigh Valley Health Network/University of South Florida Morsani College of Medicine, Bethlehem, USA

**Keywords:** emergency imaging, gerd, radiating chest pain, retention, bravo device

## Abstract

A Bravo device is a continuous pH monitor, produced by Medtronic (Minneapolis, MN), that is placed on the esophageal mucosa during endoscopy and can be used in the evaluation of gastroesophageal reflux disease (GERD). The device detaches by itself and passes with feces in approximately 7-10 days. Because of its brief presence in the body, the device is an unusual finding on imaging and could easily be mistaken for a more ominous foreign body. The Bravo device is typically well tolerated but can be a source of severe discomfort. Its presence is an important consideration in the differential of chest pain and a contraindication to MRI. Here, we discuss a case of a patient presenting to the emergency department (ED) with acute chest pain who underwent Bravo device placement several days prior.

## Introduction

Gastroesophageal reflux disease (GERD) is a chronic digestive disorder, characterized by the regurgitation of gastric contents into the esophagus, that affects millions of people worldwide [1]. Patients whose GERD symptoms are not improved by medications are recommended to undergo endoscopy with esophageal pH monitoring [2]. One such method of monitoring is the Bravo pH monitoring system, produced by Medtronic (Minneapolis, MN), which measures esophageal acid exposure [3]. This device utilizes a wireless pH sensing capsule with a complete prepackaged system and data processing software [4].

The Bravo device is not intended to be a permanent implant. Under normal conditions, the device is designed to remain in the esophageal mucosa for 48-96 hours [5]. In rare cases, patients may experience severe chest pain after Bravo device placement requiring an endoscopy for device removal [6]. It is important for physicians to recognize and understand the implications of this device during subsequent testing. We present a case in which a patient, who was diagnosed with GERD, presented to the emergency department (ED) with severe chest and arm pain following Bravo device placement.

## Case presentation

Using an interpreter, a 56-year-old female Spanish-speaking patient presented to the ED complaining of sudden-onset chest and right upper extremity pain that began the day before. In triage, the nurses assessed her and placed standard work orders for chest pain that included a posteroanterior and lateral view chest radiograph. The radiologist who reviewed the image while the patient was still in the waiting room placed a critical alert phone notification to the emergency department that there was a foreign body evident in the image. The concern was that it needed to be evaluated for the potential time-sensitive removal of a button battery device. She was immediately brought back to an examination room, and the treating physician, as part of her care, reviewed her recent outpatient medical record.

Her previous medical history was significant for GERD that was uncontrolled by maximum-dose proton pump inhibitors, right cervical radiculopathy, small vessel disease, and fibromyalgia. Three days prior to presentation, the patient had undergone an esophagogastroduodenoscopy (EGD), and the procedural report indicated that she had received a Bravo device placement and subsequent 48-hour pH study. Either from a healthcare knowledge deficit or a limitation in translator capacity, the patient was not aware that the EGD or the placement of the device was an important historical feature. On presentation, her pain was associated with nausea and dysphagia and was not relieved with acetaminophen. The patient’s initial vital signs were a blood pressure of 113/76 mmHg, pulse of 90 beats/minute, respiratory rate of 24 breaths/minute, oxygen saturation of 95%, and temperature of 36.4°C. On examination, there was tenderness to palpation present on the chest wall.

**Figure 1:**
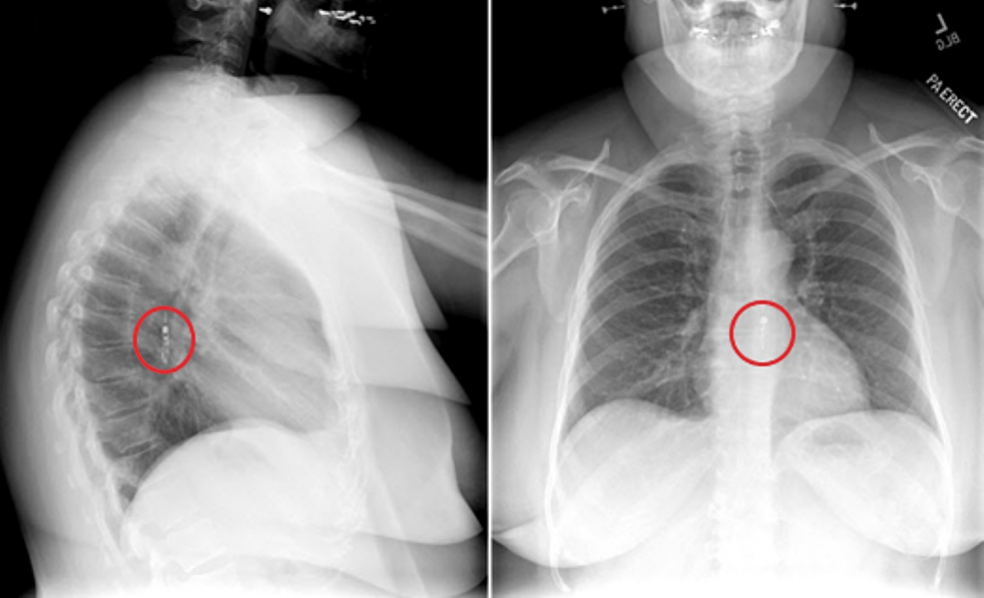
Lateral (left panel) and posteroanterior (right panel) chest X-ray images highlighting the Bravo device (red circle) placed in the patient’s esophageal mucosa three days prior.

Concerned for a possible acute coronary syndrome, in triage, an EKG and a cardiac panel were additionally ordered with standard laboratory testing. Laboratory findings were unremarkable, save for minimally elevated high-sensitivity troponin (4 ng/L; reference range: <55 ng/L). Her EKG findings were unremarkable as well. The patient was given fentanyl and hydromorphone injections to manage her pain. The final report and interpretation after conferring with the radiologist were that the 3.4 cm foreign body in the mid-esophageal region that imaging revealed was consistent with the Bravo device (Figure 1).

The chest pain was attributed to the placement of a Bravo device, and the patient was discharged with instructions to return the following day for the endoscopic removal of the retained Bravo device. When she was contacted to schedule a follow-up appointment, the patient remarked that her chest pain had resolved, and she planned to follow-up with orthopedics for her arm pain that day.

## Discussion

The Bravo device, which consists of a pH sensor and wireless transmitter, records the pH on a recorder device kept close to a patient’s body [7]. Under normal circumstances, the Bravo device sticks to the esophageal mucosa, detaches by itself, and passes through the digestive system in 7-10 days. It is important to note, however, that MRIs are contraindicated for 30 days after the placement of a Bravo device. The use of a Bravo device can be beneficial since it is usually well tolerated by patients and allows for nearly continuous pH monitoring under more physiological conditions [7]. In rare cases, device placement can aggravate the esophageal lining, causing severe discomfort [6]. The characteristics and severity of the pain can vary between patients, but it frequently mimics symptoms experienced during a cardiac event [6].

When adverse effects appear resulting from Bravo placement, it may be necessary to perform an endoscopy to remove the device before the initial study period has been completed. This case highlights the importance of accounting for various treatment and diagnostic methods used for GERD when developing a diagnostic differential. Since this is a common condition that affects millions of patients, there are a variety of management options used, and it is therefore critical to account for the function and limitations of these treatments. In this case, the patient underwent an EGD with a Bravo placement to monitor pH levels.

## Conclusions

The Bravo device is an increasingly common method of esophageal pH monitoring, which is usually well tolerated but can present with chest pain mimicking acute coronary syndrome. Because of its brief presence in the body, it is rarely seen on imaging, and it may not be recognized readily by clinicians and can therefore be easily mistaken. The awareness of its presence is important for a clinician because it can explain presenting symptoms such as chest pain, and it is an important contraindication to MRI.
